# Skeletal muscle protein turnover and mitochondrial responses to omega-3 fatty acid supplementation: an update

**DOI:** 10.1097/MCO.0000000000001196

**Published:** 2026-01-06

**Authors:** Jack E. Hayden, Colleen S. Deane

**Affiliations:** a Human Development & Health, Faculty of Medicine, University of Southampton; b NIHR Southampton Biomedical Research Centre, University Hospital Southampton NHS Foundation Trust and University of Southampton, Southampton, UK

**Keywords:** mitochondria, muscle protein synthesis, omega-3 fatty acids, skeletal muscle

## Abstract

**Purpose of review:**

To critically review recent findings related to the effects of omega-3 fatty acid supplementation on skeletal muscle, with a particular focus on skeletal muscle protein turnover and mitochondrial function.

**Recent findings:**

Evidence indicates that omega-3 fatty acids, particularly eicosapentaenoic acid (EPA) and docosahexaenoic acid (DHA), may support skeletal muscle health by influencing muscle protein synthesis (MPS), mitochondrial function, and redox balance. However, recent meta-analyses reveal inconsistent effects of omega-3 fatty acid supplementation on basal and stimulus-induced MPS, likely due to methodological variability. Omega-3 fatty acid supplementation is seemingly more beneficial in clinical cohorts and preclinical data suggests omega-3s may reduce oxidative stress.

**Summary:**

Omega-3 fatty acid supplementation is a promising nutritional strategy for supporting skeletal muscle health, via the modulation of MPS and mitochondrial function. However, large-scale trials in a variety of healthy and clinical populations using sustainable sources of omega-3 fatty acids are required before a consensus on efficacy can be made.

## INTRODUCTION

Skeletal muscle is the largest tissue in the human body, accounting for ~40% of whole-body mass in ambulatory healthy adults. In addition to producing locomotion, skeletal muscle plays critical metabolic roles in amino acid storage, lipid oxidation and glucose disposal [1,2]. Despite its fundamental role in maintaining human health, skeletal muscle mass and function rapidly decline in response to short periods of disuse such as bed rest or immobilization due to hospitalization or injury [3,4], and progressively decline during chronological ageing (sarcopenia) [5]. Losses in muscle mass and function lead to negative metabolic outcomes including impaired protein turnover, and insulin resistance [6], and compound over time increasing the risk of falls, frailty and overall mortality. As such, identifying interventions aimed at offsetting skeletal muscle decline and maintaining muscle health across the life course is a growing socioeconomic and clinical priority.

Omega-3 (*n*-3) polyunsaturated fatty acids are a class of long chain fatty acids, found abundantly in oily fish (e.g., salmon, tuna) [7^▪^], which have positive associations and impacts on skeletal muscle health [8–12]. The major bioactive omega-3 fatty acids, eicosapentaenoic acid (EPA) and docosahexaenoic acid (DHA), have been implicated in preserving skeletal muscle primarily via their effects on muscle protein synthesis (MPS) [13^▪▪^] and mitochondrial adaptations [14,15]. In this brief review, we provide an update on the impact supplemental omega-3 fatty acids have on skeletal muscle, with a particular focus on protein turnover and mitochondrial function, building on prior work in this area [9]. Due to the brevity of this review, not all interactions between omega-3 and skeletal muscle have been covered, and so readers are referred to recent relevant reviews on the topic (e.g., [10,16]). 

**Box 1:**
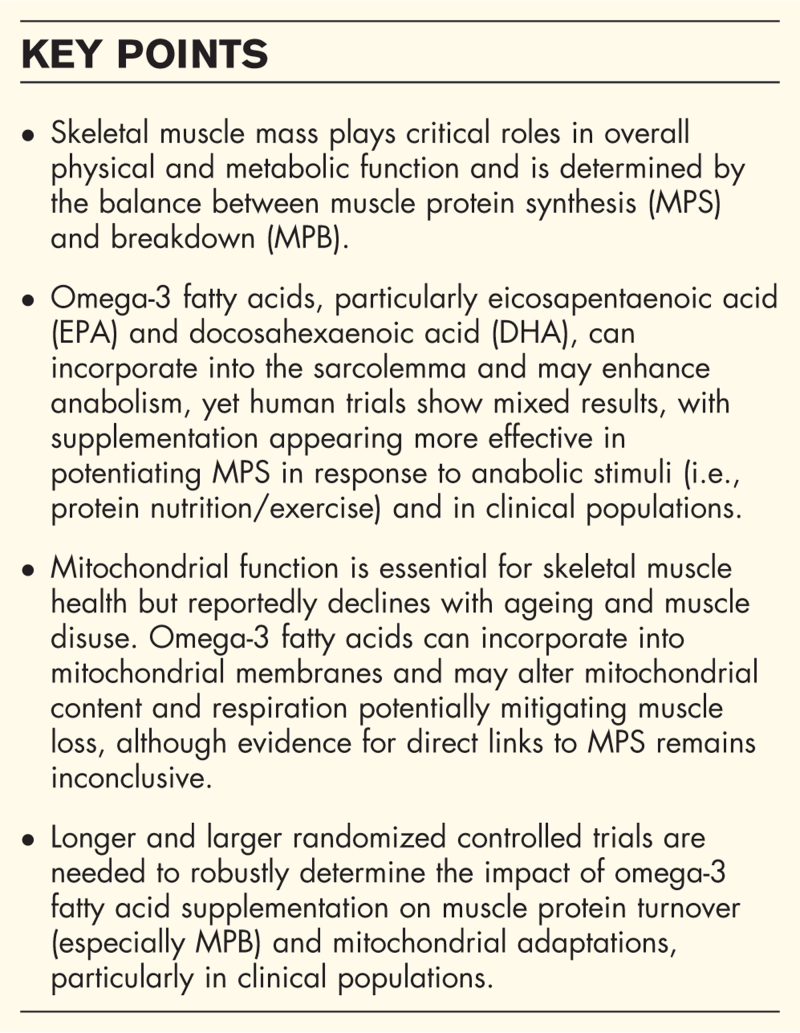
no caption available

## OMEGA-3 FATTY ACIDS AND SKELETAL MUSCLE PROTEIN TURNOVER

Skeletal muscle mass is governed by the dynamic balance between MPS and muscle protein breakdown (MPB) [17]. In the rested and fasted (postabsorptive) state, MPB exceeds MPS leading to a transient net negative protein balance [17], whereas in the fed (postprandial) state i.e., in response to a protein/essential amino acid-rich meal, MPS is stimulated and exceeds MPB, resulting in a transient positive protein balance [17]. This diurnal dynamism ensures that muscle mass remains constant in healthy ambulatory adults. However, during a period of disuse, rates of MPS significantly decrease in both fasted and fed states leading to muscle atrophy/sarcopenia [3]. MPB also declines albeit at a much lesser magnitude and thus declines in MPS are considered the driving force of muscle atrophy/sarcopenia (reviewed extensively in [3]). While the provision of a protein-rich meal/supplement can maximally stimulate MPS [18], dietary protein can be inadequate in isolation to offset disuse-induced muscle atrophy and age-related sarcopenia, requiring adjunct nutritional interventions that support protein turnover in these scenarios. Considering MPS is the primary anabolic driver and given the methodological challenges around measuring MPB [19], herein we focus on the regulation of MPS only.

**FIGURE 1:**
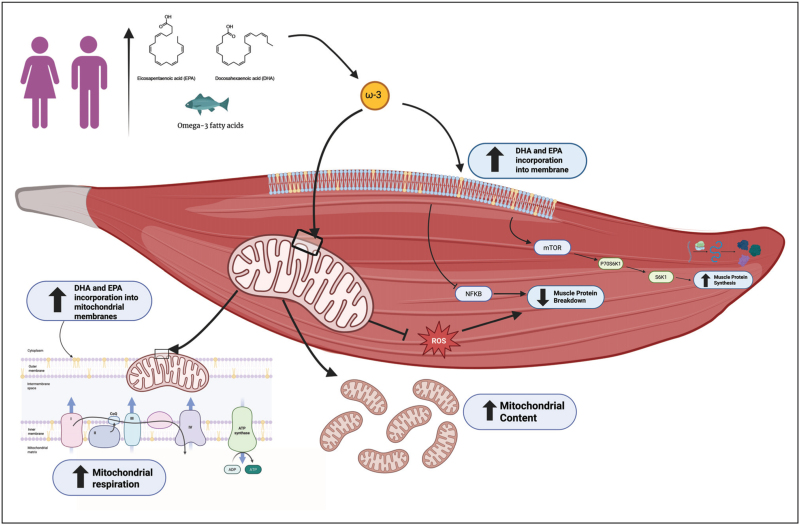
Benefits of omega-3 fatty acid supplementation on protein metabolism and mitochondria function in skeletal muscle. Dietary omega-3 fatty acid supplementation leads to the incorporation of eicosapentaenoic acid (EPA) and docosahexaenoic acid (DHA) into skeletal muscle and mitochondrial membranes. This incorporation may trigger downstream signalling through mechanistic target of rapamycin (mTOR) and nuclear factor kappa B (NFĸB), regulating muscle protein synthesis and breakdown, respectively, and may regulate mitochondrial content, respiration and reactive oxygen species (ROS).

Omega-3 fatty acids, widely recognized for their cardiometabolic benefits [20], have been shown to enhance muscle mass and strength in healthy and clinical populations [10,21], although this has been recently debated [22], pointing to a potential role in promoting muscle anabolism [13^▪▪^]. Mechanistically, omega-3 fatty acids readily incorporate into the phospholipid membranes of skeletal muscle cells [23^▪▪^], where they may upregulate molecular signalling pathways that drive muscle growth and remodelling (i.e., mTOR signalling) (Fig. 1) [13^▪▪^]. However, despite the biological action and plausible rationale, experimental findings on the anabolic effects of omega-3 fatty acid supplementation remain mixed [13^▪▪^,^24^^▪▪^]. To clarify the conflicting results, a recent meta-analysis by Therdyothin *et al.* [24^▪▪^] synthesized data from the relevant human literature. The authors reported no effect of omega-3 fatty acids on basal MPS in healthy adults regardless of dose/duration of supplementation, participant age or methodology used to determine synthetic rates, despite several of the original studies reporting increases in muscle mass [24^▪▪^]. While no robust basal changes in MPS were evident, omega-3 fatty acid supplementation is thought to potentiate MPS in response to anabolic stimuli (i.e., protein nutrition/exercise). Evidencing this, early seminal work by Smith *et al.* demonstrated that omega-3 fatty acid supplementation, while not impacting basal MPS, increased the anabolic response to a hyperaminoacidemia-hyperinsulinemia clamp in young and middle-aged males and females [25], with similar findings reported in some clinical cohorts [26]. However, the recent meta-analysis by Therdyothin *et al.* failed to detect an increase in MPS in response to omega-3 fatty acid supplementation adjuvant to an anabolic stimulus [24^▪▪^]. While perhaps unexpected, the heterogeneity in study methodologies may account for this null finding. For example, studies included in the meta-analysis employed different forms of anabolic stimuli (insulin and amino acid infusion versus exercise), collected muscle tissue biopsies at different timepoints (ranging from 1 to 4 h post anabolic stimulus) and may have achieved maximal MPS with anabolic stimulus alone rendering any additional benefits of omega-3 fatty acids undetectable [24^▪▪^]. Moreover, the number of participants per trial was low and individual variation in the MPS response to omega-3 fatty acids was high, also likely contributing to the null finding. It is worth noting that while no effects on MPS were detected, omega-3 fatty acids did lead to significant increases in whole body-synthesis rates, particularly in clinical populations (cachexia/chronic inflammation), suggesting that omega-3 fatty acids may be more beneficial in these cohorts. Therefore, larger controlled trials are warranted to robustly demonstrate whether omega-3 fatty acid supplementation impacts MPS in both healthy adults and in those characterized by muscle decline.

## OMEGA-3 FATTY ACIDS AND SKELETAL MUSCLE MITOCHONDRIA

Mitochondria contribute to skeletal muscle health and homeostasis via roles in ATP production, reactive oxygen species (ROS) and antioxidant regulation, calcium handling, and apoptosis. In healthy adults, mitochondria account for ~4–7% of muscle cell volume and significantly correlate with physical capacity [27,28]. However, in response to disuse and ageing, mitochondrial content and respiration tend to decline [27] (although debated), which can translate into impaired maximal ATP production and lowered fatty acid β-oxidation with subsequent glucose intolerance and impaired respiratory capacity [29], thus requiring novel mitochondria-targeting countermeasures.

In addition to incorporating into the sarcolemma [23^▪▪^], omega-3 fatty acid supplementation leads to increased EPA and DHA content in human skeletal muscle mitochondrial membranes, alongside improved ADP sensitivity [30], suggesting an important role for omega-3s in regulating mitochondria (Fig. 1). Subsequent work has shown omega-3 fatty acids attenuate disuse-induced declines in skeletal muscle mitochondrial content and respiration, alongside reduced declines in muscle mass, suggesting a mechanistic link between mitochondrial content/function and MPS (reviewed in [9]). This has led to the hypothesis that the incorporation of omega-3 fatty acids into mitochondrial membranes, particularly during pathological scenarios such as disuse, will improve aspects of mitochondrial respiration therein maintaining mitochondrial protein transcription and translation. Sustained mitochondrial protein translation then permits signals to the translational machinery within the sarcoplasm to support MPS so mitigating declines in muscle mass [9]. While an intriguing and plausible hypothesis worthy of future investigation, recent evidence has shown that mitochondrial bioenergetics are not associated with MPS rates in healthy ambulatory adults or in adults who have undergone an acute period of disuse [31]. Thus, future work is needed to explore the crosstalk between mitochondrial bioenergetics and MPS in the context of omega-3 fatty acid exposure.

Omega-3 fatty acids are also well known for their role in redox homeostasis, which intersects with mitochondrial health through the ability to modulate ROS production, activate antioxidant pathways and enhance mitochondrial quality-control processes. In this context, recent work in sarcopenic mice found that EPA and DHA both reduced age-related oxidative stress in skeletal muscle, with EPA exerting a more pronounced ROS-lowering effect [32^▪^]. This improved redox environment was closely linked to more favourable muscle phenotypes whereby lower oxidative damage was accompanied by reduced activation of atrophy-related pathways, enhanced PI3K/Akt/mTOR signalling and greater preservation of fibre size and strength [32^▪^]. Thus, redox regulation may be a central mechanism by which omega-3 fatty acids can mitigate age-related muscle decline (Fig. 1).

## FUTURE DIRECTIONS

As research into omega-3 fatty acids and skeletal muscle health continues to evolve, several promising avenues warrant further exploration. First, there is a clear need to conduct large-scale, well controlled trials to robustly elucidate the effects of omega-3 fatty acid supplementation on MPS, MPB, mitochondrial and muscle mass/function. Effectiveness likely depends on the dose, duration of supplementation, age, physical (in)activity status and health status, yet these factors remain poorly defined and inconsistently controlled across studies. Carefully designed trials that stratify or directly test these variables are necessary to unravel the true efficacy and context-specific responsiveness of omega-3 fatty acids in skeletal muscle. Second, findings from young, healthy ambulatory adults – who typically have intact protein turnover and mitochondrial function – may not translate to individuals characterized by muscle wasting (e.g., sarcopenia/disease/critical care), where anabolic resistance and mitochondrial dysfunction are pronounced. Such physiological differences are likely to also alter omega-3 fatty acid uptake, utilization and efficacy. Distinguishing the effects of supplementation in cohorts characterized by muscle loss is therefore essential, as responses in healthy muscle may underestimate the potential therapeutic impact of omega-3 fatty acids in these more vulnerable populations.

Third, future studies should investigate the sexual dimorphic response to omega-3 fatty acid supplementation since the anabolic response is purported to be more pronounced in females (compared to males) [23^▪▪^,^24^^▪▪^], which may be due to greater skeletal muscle EPA phospholipid content in women following omega-3 fatty acid supplementation [23^▪▪^]. Fourth, to advance our understanding of the molecular mechanisms by which omega-3 fatty acids impact skeletal muscle, future studies should apply and integrate multiple omics technologies (i.e., transcriptomics, proteomics, lipidomics, and metabolomics) which, in addition to revealing complex regulatory networks, could also identify novel biomarkers of response, and identify how different omega-3 fatty acid sources impact molecular pathways related to muscle protein/mitochondrial metabolism. This approach would ultimately support the development of more targeted, personalized, and sustainable omega-3 fatty acid-based strategies for muscle health.

Finally, as global demand for omega-3 fatty acids increases, the need for sustainable sourcing needs to be considered [7^▪^,^33^]. Nonfish derived sources of EPA and DHA are already available and include different microalgae species, algal oils, and genetically modified oilseed crops, which display similar bioavailability and cardiovascular impacts compared to fish oil (reviewed in [7^▪^]). Additionally, many plants, such as green leaves, seeds and nuts, contain alpha linolenic acid (ALA) and plant seed oils, such as *Buglossoides arvensis* (Ahiflower), contain stearidonic acid (SDA), which are precursors of EPA and have been shown to improve markers of whole-body and hepatic glucose metabolism (reviewed in [7^▪^]). Despite the potential of these alternative sources to mimic the metabolic impacts of fish-derived omega-3 fatty acids on markers of whole-body health, their bioavailability within muscle and their direct effects on muscle protein turnover and mitochondrial function remain to be fully determined.

## CONCLUSION

Collectively, current evidence suggests that omega-3 fatty acids hold promise as an adjunct nutritional strategy to support skeletal muscle health, particularly through their proposed roles in modulating MPS and mitochondrial function (Fig. 1). While mechanistic data highlight the ability of EPA and DHA to incorporate into muscle and mitochondrial membranes, influence MPS and mitochondrial respiration, especially during periods of disuse, findings from human trials remain inconsistent. Variability in study design, participant characteristics, and methodological approaches currently preclude consensus regarding their anabolic potential. Nevertheless, emerging insights into whole-body protein turnover, redox regulation and potential sex-specific responses demonstrate the relevance, and complexity, of omega-3 fatty acid supplementation in skeletal muscle biology. Moving forward, rigorous, large-scale trials paired with integrative omics approaches and sustainable omega-3 fatty acid sourcing will be essential to clarify their efficacy and optimise personalized strategies.

## Acknowledgements

*None.*

### Financial support and sponsorship

*None to declare.*

### Conflicts of interest

*There are no conflicts of interest.*

## REFERENCES AND RECOMMENDED READING

Papers of particular interest, published within the annual period of review, have been highlighted as:

▪ of special interest
▪▪ of outstanding interest
